# Association between furosemide in premature infants and sensorineural hearing loss and nephrocalcinosis: a systematic review

**DOI:** 10.1186/s40748-018-0092-2

**Published:** 2018-11-19

**Authors:** Wesley Jackson, Genevieve Taylor, David Selewski, P. Brian Smith, Sue Tolleson-Rinehart, Matthew M. Laughon

**Affiliations:** 1 Division of Pediatrics, University of North Carolina at Chapel Hill, UNC Hospitals 101 Manning Dr. 4th Floor, Chapel Hill, NC CB 7596 USA; 2 Division of Nephrology, Department of Pediatrics and Communicable Diseases, C.S. Mott Children’s Hospital, University of Michigan, Ann Arbor, MI USA; 3 Duke Department of Pediatrics, Duke University Medical Center, Durham, NC USA; 4 Gillings School of Global Public Health, University of North Carolina at Chapel Hill, Chapel Hill, USA

**Keywords:** Furosemide, Infant, Premature, Sensorineural hearing loss, Nephrocalcinosis, Nephrolithiasis

## Abstract

Furosemide is a potent loop diuretic commonly and variably used by neonatologists to improve oxygenation and lung compliance in premature infants. There are several safety concerns with use of furosemide in premature infants, specifically the risk of sensorineural hearing loss (SNHL), and nephrocalcinosis/nephrolithiasis (NC/NL). We conducted a systematic review of all trials and observational studies examining the association between these outcomes with exposure to furosemide in premature infants.

We searched MEDLINE, EMBASE, CINAHL, and clinicaltrials.gov. We included studies reporting either SNHL or NC/NL in premature infants (< 37 weeks completed gestational age) who received at least one dose of enteral or intravenous furosemide. Thirty-two studies met full inclusion criteria for the review, including 12 studies examining SNHL and 20 studies examining NC/NL. Only one randomized controlled trial was identified in this review. We found no evidence that furosemide exposure increases the risk of SNHL or NC/NL in premature infants, with varying quality of studies and found the strength of evidence for both outcomes to be low. The most common limitation in these studies was the lack of control for confounding factors.

The evidence for the risk of SNHL and NC/NL in premature infants exposed to furosemide is low. Further randomized controlled trials of furosemide in premature infants are urgently needed to adequately assess the risk of SNHL and NC/NL, provide evidence for improved FDA labeling, and promote safer prescribing practices.

**Electronic supplementary material:**

The online version of this article (10.1186/s40748-018-0092-2) contains supplementary material, which is available to authorized users.

## Background

Furosemide is a potent diuretic that acts in the proximal and distal tubules, as well as the loop of Henle, to inhibit sodium and chloride reabsorption in the kidneys. The use of diuretics such as furosemide may alleviate symptoms associated with volume overload, including pulmonary edema. In premature infants, early pulmonary edema and excessive intravenous fluid administration are associated with an increased risk of bronchopulmonary dysplasia (BPD), also called chronic lung disease of prematurity [1, 2]. Therefore, furosemide may be part of a clinical approach to reducing the risk of BPD in premature infants.

The Food and Drug Administration (FDA) has approved furosemide for the treatment of edema associated with congestive heart failure, cirrhosis, and nephrotic syndrome in children and adults. Furosemide is not approved by the FDA for use in premature infants and, as a result, any use in this population is considered off-label. Specifically, the FDA label for furosemide includes a warning that infants < 31 weeks postmenstrual age receiving doses > 1 mg/kg/day intravenously may develop plasma levels resulting in ototoxicity. The label also notes that renal function monitoring and renal sonography should be considered in premature infants, as furosemide may precipitate nephrocalcinosis.

The primary safety concern of furosemide use in premature infants is sensorineural hearing loss (SNHL). The incidence of SNHL is approximately 0.7–1.5% among infants admitted to the NICU and is more common in infants born prematurely [3, 4]. The association of SNHL and furosemide relies heavily on studies conducted in adults receiving high doses of furosemide. In one study, reversible hearing loss occurred in 50% of adult patients with uremia given a single 1000 mg intravenous dose of furosemide, which is 50 times the usual adult dose [5]. A case series reported transient deafness in 3 adult patients with renal impairment given intravenous doses of 2000–3000 mg [6]. Finally, a trial of 19 adult patients receiving furosemide or placebo resulted in one patient with permanent deafness who had received 14 days of 1000 mg per day. The authors of the trial reported that furosemide levels > 100 μg/mL were associated with ototoxicity [7]. The proposed mechanisms for furosemide-induced ototoxicity include changes in potassium concentrations in the cochlear endolymph and impairment of cellular proliferation [8, 9].

Furosemide use in premature infants has also been implicated in the development of nephrocalcinosis and nephrolithiasis, or renal calcifications and stones, although the etiology in this population is likely to be multifactorial [10, 11]. Nephrocalcinosis is diagnosed by renal ultrasonography and has the appearance of increased echogenicity in the medullary pyramids of the kidney. Renal calculi, or nephrolithiasis, are detected on ultrasound by echogenic foci in the calyces or renal pelvis. Loop diuretics, such as furosemide, reduce renal tubular reabsorption of calcium resulting in hypercalciuria, which is the proposed mechanism for its association with nephrocalcinosis. However, this association is often confounded by concomitant exposure to other therapies, such as dexamethasone, long-term parenteral nutrition, and mechanical ventilation, which have been identified as risk factors for renal calcifications [12, 13].

In the absence of robust data from large, well-powered clinical trials, the best available method for evaluating the safety of furosemide in premature infants includes a thorough review of the limited number of randomized control trials, as well as non-controlled studies such as cohort and case-control studies. This systematic review will synthesize all trials and observational studies in which premature infants were exposed to at least one dose of furosemide and report on the following outcomes: SNHL and nephrocalcinosis/nephrolithiasis (NC/NL). This review differs from Cochrane reviews in its inclusion of observational studies, which comprise the majority of available evidence for furosemide safety in premature infants. The key questions this systematic review will address are the following:

- *Key Question 1*: Does exposure to furosemide in premature infants increase the risk of SNHL?
- *Key Question 2*: Does exposure to furosemide in premature infants increase the risk of NC/NL?

## Methods

We reviewed all observational cohort studies or clinical trials in which premature infants (< 37 weeks completed gestational age) were exposed to at least one dose of furosemide while hospitalized in the NICU. We used premature infants without exposure to furosemide, when available, as comparators. The outcomes of interest were SNHL and NC/NL. Table 1 displays the eligibility criteria for this systematic review.

**Table 1:** Eligibility Criteria

Patient/Population	Infant < 37 weeks completed gestational age
Intervention	≥1 dose of furosemide (IV or PO) during hospitalization in the neonatal intensive care unit
Control	Infant < 37 weeks completed gestational age without exposure to furosemide
Outcomes	sensorineural hearing loss; nephrocalcinosis/nephrolithiasis
Study Design	clinical trials, retrospective or prospective cohort studies, case-control studies

We searched the following databases for the relevant literature published in English: MEDLINE, EMBASE, and CINAHL. We also searched clinicaltrials.gov to include results of unpublished studies. The references of relevant articles within this search were reviewed by the investigators for additional articles of interest. The date of the most recent search was February 3, 2018. The MEDLINE search used the following Medical Subject Heading terms: “furosemide” AND “infant, premature.” The final search included the following string of search terms: “furosemide”[MeSH Terms] OR “furosemide”[All Fields] AND “infant, premature”[MeSH Terms] OR (“infant”[All Fields] AND “premature”[All Fields]) OR “premature infant”[All Fields] OR “preterm”[All Fields] AND “infant”[All Fields] OR “preterm infant”[All Fields] OR “neonate”[All Fields]. The EMBASE and CINAHL searches used the following terms: (‘premature infant’/exp. OR ‘premature infant’ OR ((‘premature’/exp. OR premature) AND (‘infant’/exp. OR infant))) AND (‘furosemide’/exp. OR furosemide). The clinicaltrials.gov search used the terms “premature infant” and “furosemide.”

We compiled all studies resulting from the preceding search strategy and removed duplicates. Two authors (WJ and GT) reviewed titles and abstracts for relevance using the software program Abstrackr [14]. We screened full text articles and developed data abstraction forms to determine eligibility of each study. If eligibility criteria were met, we recorded the study design, population characteristics, characteristics of comparison group, outcomes examined, sample size, duration of follow-up, and funding sources. These data abstraction forms were used by the investigators to report on the results (Additional file 1).

We assessed the quality (internal validity) of randomized controlled trials using the Cochrane Collaboration’s tool for assessing risk of bias in clinical trials [15]. This tool includes the assessment of randomization procedures, allocation concealment, blinding of participants and investigators, completeness of outcomes data, selective reporting, and the risk of other biases. Each randomized controlled trial included in the systematic review was graded on each of these parameters as high risk of bias, low risk of bias, or unclear risk of bias. We assessed the risk of bias in non-randomized studies using the ROBINS-1 tool, which assesses confounding biases, bias in the selection of participants, classification biases, biases due to deviations from intended interventions, missing data biases, and biases in measurement of outcomes [16]. Bias is rated on a scale of low, moderate, serious, or critical risk of bias, or no information. We then graded the strength of the evidence for each key question using guidance from the Evidence-based Practice Center program of the U.S. Agency for Healthcare Research and Quality [17].

The pre-specified outcome measures were odds ratios and risk ratios, where appropriate, with 95% confidence intervals for the exposure of furosemide in premature infants with each of the outcomes of interest addressed in the key questions. Results of each study were described and only the information pertaining to the outcomes of interest in the key questions were included.

## Results

**Fig. 1:**
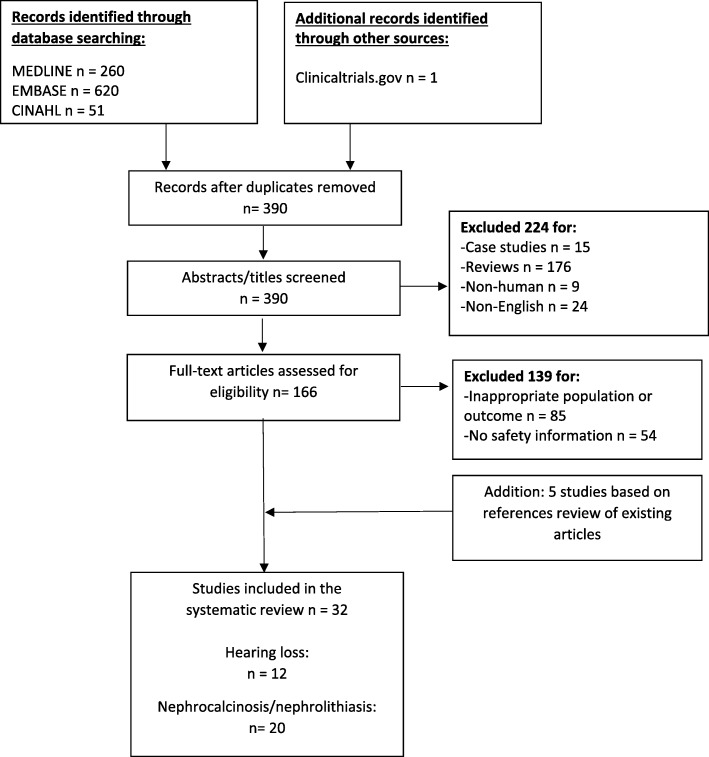
PRISMA flow diagram for inclusion and exclusion of studies [71]

We identified 260 records through MEDLINE, 620 records through EMBASE, 51 records from CINAHL, and 1 study from clinicaltrials.gov. After removing duplicates, we screened 390 records for eligibility. We excluded 224 records due to study design, non-human population, or non-English language. We reviewed the full text of the remaining 166 articles and excluded 139 articles for inappropriate study population or lack of relevant safety outcome. We identified 5 articles in the reference lists of reviewed articles which met the eligibility criteria. These studies did not appear in the original search due to the use of a variation in the drug name: “frusemide”, instead of “furosemide.” In total, 32 articles were included in the quantitative analysis. See Fig. 1 for the PRISMA flow diagram.

### Key question 1: Does exposure to furosemide in premature infants increase the risk of sensorineural hearing loss?

Only one randomized controlled trial of furosemide compared to placebo in premature infants reported on the outcome of SNHL [18]. In this trial, 24 premature infants were randomized to receive either furosemide or placebo for 7 days. The initial dose of furosemide (1 mg/kg IV or 2 mg/kg orally every 12 h) was doubled after 48 h in infants where there was not a 50% increase in urine output over 12 h. In the 17 infants available for analysis (7 infants in treatment group and 10 infants in the control group), no SNHL was detected prior to discharge.

We identified 11 observational studies examining the association of furosemide and SNHL in premature infants: 5 retrospective cohort studies and 6 case-control studies [19–29]. The results of the studies were mixed; however, an association between furosemide and SNHL was found in 8 of the 11 observational studies [19–23, 25, 27, 29]. Two cohort studies and one case-control study did not identify an association with furosemide and SNHL [24, 26, 28]. These negative studies were conducted in single centers and did not consider dose exposure of furosemide.

Table 2 summarizes the population characteristics, sample size, outcome measures, and results from each study. The studies varied considerably in their definitions of SNHL, including the type of auditory testing used and length of follow-up. However, every study included a hearing screen in all infants prior to NICU discharge and every infant classified as having SNHL failed the initial newborn hearing screen. Auditory testing in these studies included either auditory brain stem response (ABR) [19, 20, 22, 28], brainstem auditory evoked response (BAER) [21], otoacoustic emission (OAE) test [23], behavioral audiometry in older children, or some combination of these tests [24–27, 29]. Several studies differentiated auditory neuropathy or auditory neuropathy spectrum disorders, in which there is a defect in the transmission of sound from the inner hair cells of the cochlea to the brain via the auditory nerve, from the broader category of SNHL [25–27]. Infants with auditory neuropathy have an abnormal ABR with preserved OAE testing, while those with SNHL have abnormal results on both tests.

**Table 2:** Summary of studies examining risk of hearing loss in premature infants

Study (Year)	Design	Population and Sample Size	Outcome Measure	Results
Mjoen (1982) [19]	cohort	60 high-risk infants 27–44 weeks GA	ABR testing in NICU and follow-up visits	• 4 infants with evidence of SNHL. • 3/4 infants exposed to ototoxic medications (furosemide and/or aminoglycoside).
McCann (1985) [18]	randomized controlled	17 premature infants with BPD (7 infants received furosemide and 10 infants received placebo)	Audiology screen at discharge and follow-up visits	• Normal hearing in all infants.
Salamy (1989) [20]	cohort	GA 24–34 weeks	ABR in NICU and follow-up; behavioral audiometry from 3 months to 4 years	• Infants with SNHL received greater amounts of furosemide for longer durations, in combination with aminoglycoside or vancomycin therapy (*p* < 0.001 for all factors).
Brown (1991) [21]	case-control	35 infants with SNHL and 70 matched hearing-intact controls	BAER testing prior to discharge from NICU	• 17/35 (49%) infants with SNHL and 6/70 (9%) controls were exposed to furosemide (*p* < 0.0001).
Borradori (1997) [22]	case-control	8 children with progressive bilateral deafness born preterm (GA ≤ 34 weeks) with 16 controls matched on GA and BW and 15 controls matched on perinatal complications	ABR at NICU discharge and follow-up	• 8/8 (100%) infants with SNHL and 13/15 (87%) controls received furosemide (NS). • Mean duration (p < 0.001), total cumulative dose (p < 0.001), and maximum daily dose (*p* = 0.05) were higher in SNHL group.
Ertl (2001) [23]	case-control	22 premature infants with SNHL and 25 controls matched on GA, BW, and perinatal factors associated with hearing loss	OAE test and ABR if failed OAE	• 4/22 (18%) infants with SNHL and 1/25 (4%) controls received furosemide (p < 0.01).
Rais-Bahrami (2004) [24]	cohort	57 infants who received furosemide and 207 infants who did not receive furosemide	OAE, ABR, or both prior to NICU discharge	• No difference in abnormal hearing screen in furosemide and non-furosemide groups (16% vs. 16%; *p* = 0.95).
Xoinis (2007) [25]	case-control	71 infants with SNHL, 24 with auditory neuropathy,and 95 controls matched on GA, BW, and birth year	ABR and OAE	• Higher exposure to furosemide in SNHL group (51%) and AN group (96%) compared to control group (32.6%) (*p* < 0.05) for both comparisons.
Coenraad (2011) [26]	case-control	9 infants with hearing loss and 36 controls matched on GA, gender, and birth year	ABR screening prior to NICU discharge and repeat ABR and OAE at follow-up visit for failed screening.	• No differences in furosemide exposure between groups (44% vs. 25%; *p* = 0.56).
Martinez-Cruz (2012) [27]	case-control	6 children with SNHL and 87 normal-hearing controls with birth weights < 750 g	BAER screening and OAE at follow-up visits for failed initial screening	• 6/6 (100%) infants with SNHL and 45/87 (52%) control infants received furosemide (*p* = 0.002). • Longer average duration of furosemide in SNHL infants who received furosemide compared with controls (18 days vs. 7 days).
Rastogi (2013) [28]	cohort	Infants with BW < 1500 g.	ABR prior to NICU discharge; Follow-up at 2 years for failed screening to determine hearing status	• No association with furosemide and hearing loss when adjusting for BW, GA, and other perinatal risk factors (OR 1.18; *p* = 0.3).
Wang (2017) [29]	cohort	Included all infants with BW ≤ 1500 g. 297 infants with normal hearing and 12 infants with hearing loss	OAE before discharge and BAER at 3 months corrected age if failed initial screen	• Exposure to ototoxins (furosemide and/or gentamicin) was associated with hearing loss (OR 3.62; 95% CI 1.67–7.82).

Legend: *GA* Gestational Age, *ABR* Auditory brainstem response, *BPD* Bronchopulmonary dysplasia, *SNHL* Sensorineural hearing loss, *BAER* Brainstem auditory evoked response, *NICU* Neonatal Intensive Care Unit, *BW* Birthweight, *NS* Non-significant, *OAE* Otoacoustic emission, *AN* Auditory neuropathy, *ANSD* Auditory neuropathy spectrum disorder, *OR* Odds ratio, *CI* Confidence Interval

Although a majority of the studies did not consider dosing, three studies described the dose and duration of furosemide and its association with SNHL and each of these studies found a positive association between furosemide and SNHL [20, 22, 27]. One found higher cumulative dose exposure of furosemide in infants with hearing loss than was true in infants without hearing loss (mean +/− standard deviation: 139.1 +/− 130 mg/kg vs. 41.5 +/− 76 mg/kg; *p* < 0.001), in addition to longer duration of furosemide use in the hearing loss group (52.5 +/− 43 days vs. 19 +/− 23 days; *p* < 0.001) [20]. In another study, investigators described higher maximum daily dose of furosemide (3.2 +/− 0.58 mg/kg vs. 2.45 +/− 0.79 mg/kg; *p* = 0.05), longer duration of treatment (17 +/− 8.3 days vs. 3.4 +/− 2.1 days; *p* < 0.001), and higher cumulative dose exposures (26.9 +/− 13.7 mg/kg vs. 6.17 +/− 4 mg/kg; p < 0.001) in infants with SNHL than in infants with normal hearing [22]. A third study reported a longer duration of furosemide treatment in infants with SNHL compared to those with normal hearing (17.5 +/− 10 days vs. 7 +/− 4.9 days; *p* = 0.002) [27].

#### Quality assessment

We assessed the quality of each study based on the risk of bias in the seven domains of the ROBINS-I tool for non-randomized studies (Table 3). The risk of bias in the McCann study is included in Table 4 using the Cochrane Risk of Bias Tool. The primary determining factor for the assessment of moderate or serious risk of bias was whether an appropriate analysis method was used that controlled for important perinatal factors, such as severity of illness, duration of hospitalization, birth weight, gestational age, and co-morbidities known to be risk factors for hearing loss. We considered two studies to have a critical risk of bias due to grouping furosemide exposure with other medications as a single variable termed “ototoxins” [19, 29]. There was no pattern relating quality to the results of the studies (i.e., both positive and negative studies included a mix of moderate and serious risks of bias).

**Table 3:** Quality assessment of observational studies examining risk of hearing loss in premature infants

Study (Year)	Risk of Bias (Low, Moderate, Serious, Critical, No Information)	Comments
Mjoen (1982) [19]	Critical	Critical risk of bias in classification of interventions domain: ototoxic medications grouped as one variable (i.e., furosemide not identified as a single risk factor).
Salamy (1989) [20]	Moderate	Confounding well-accounted for by assessing “neonatal status” based on duration of hospitalization, days of assisted ventilation, radiography and lab results, etc.
Brown (1991) [21]	Serious	Serious risk of bias in confounding domain: Selection of variables included in the multivariate analyses based solely on results of univariate analyses and did not adequately account for severity of illness in each group.
Borradori (1997) [22]	Moderate	Confounding well-accounted for by the creation of two control groups based on BW/GA and perinatal complications related to risk of ototoxicity.
Ertl (2001) [23]	Serious	Serious risk of bias in confounding domain: infants not matched on severity of illness or co-morbidities associated with hearing loss.
Rais-Bahrami (2004) [24]	Serious	Serious risk of bias in confounding domain: no adjustment for perinatal factors related to hearing loss.
Xoinis (2007) [25]	Serious	Serious risk of bias in confounding domain: infants not matched on severity of illness or co-morbidities associated with hearing loss.
Coenraad (2011) [26]	Serious	Serious risk of bias in confounding domain: infants not matched on severity of illness or co-morbidities associated with hearing loss.
Martinez-Cruz (2012) [27]	Serious	Serious risk of bias in confounding domain: infants not matched on severity of illness or co-morbidities associated with hearing loss.
Rastogi (2013) [28]	Moderate	Confounding well-accounted for in multivariate analyses, which adjusted for GA, BW, and other known perinatal risk factors for hearing loss.
Wang (2017) [29]	Critical	Critical risk of bias in classification of interventions domain: ototoxic medications grouped as one variable (i.e., furosemide not identified as a single risk factor).

Legend: *BW* Birth weight, *GA* Gestational age

**Table 4:** Risk of bias in trials examining risk of hearing loss in premature infants

Study (Year)	Risk of Bias (High, Low, Unclear)						
	Random Sequence Generation	Allocation Concealment	Blinding of Participants and Personnel	Blinding of Outcome Assessment	Incomplete Outcome Data	Selective Reporting	Other Bias
McCann (1985) [18]	Low	Low	Low	Low	High	Unclear	Low

#### Strength of evidence

We determined that the strength of evidence for the association of SNHL and furosemide exposure in premature infants is low based on our review of the existing literature. This judgement is based on the high risk of bias in the observational studies, which often did not adequately account for the severity of illness in infants exposed to furosemide, and the inconsistency in the results. Furthermore, there is a problem with the directness of comparisons as infants exposed to furosemide are also more likely to receive additional interventions which may increase the risk of SNHL, such as concomitant ototoxic medications and mechanical ventilation. According to the Agency for Healthcare Research and Quality (AHRQ), a low grade indicates “low confidence that the evidence reflects the true effect [and] further research is likely to change the confidence in the estimate of effect and is likely to change the estimate” [17]. It is clear that further clinical trials are needed to adequately assess the risk of SNHL in premature infants exposed to furosemide.

### Key question 2: Does exposure to furosemide in premature infants increase the risk of nephrocalcinosis/nephrolithiasis?

No randomized controlled trials of furosemide in premature infants have been performed that include outcome data on the incidence of NC/NL. We identified 20 cohort studies examining the association of furosemide exposure and NC/NL [30–49]. The results of the studies were mixed; however, 12 of the 20 studies found an association between furosemide and NC/NL [30–32, 34–37, 40, 41, 44, 46, 47, 49]. All studies were performed at single centers, except one, which included infants from two centers [40]. Three of the studies resembled case series in that there was no inclusion of adequate control groups without NC: one of these studies found an association of furosemide with NC and two studies did not [30, 38, 42].

Table 5 summarizes the population characteristics, sample size, outcome measures, and results from each study. There was considerable variability in the inclusion criteria, the timing of renal ultrasonography, and duration of long-term follow-up. However, all of the reviewed studies included premature infants with sonographic evidence of NC/NL prior to discharge from the NICU. Of the studies that included subsequent ultrasounds after NICU discharge, complete resolution of NC occurred in 44–100% of infants by 2 years of age [31, 33, 35, 36, 38, 39, 42, 46–49]. However, these data on outcomes are incomplete, as they do not include infants with the most severe disease, who expired during their NICU hospitalization, and infants lost to follow-up after discharge.

**Table 5:** Summary of studies examining risk of nephrocalcinosis/nephrolithiasis (NC/NL) in premature infants

Study (Year)	Design	Population and Sample Size	Outcome Measure	Results Summary
Hufnagle (1982) [30]	cohort	10 premature infants with NC	RUS during NICU admission	• All infants received furosemide of at least 2 mg/kg/day for at least 12 days prior to NC.
Woolfield (1988) [31]	cohort	36 infants with BW ≤ 1500 g	RUS at 12 months of age	• 3/32 (9%) infants had NC on RUS and had received chronic furosemide with doses ranging from 2 to 8 mg/kg/day. • NC resolved in 2/3 (67%) cases; 1 died of unrelated causes.
Jacinto (1988) [32]	cohort	31 infants with BW < 1500 g	RUS in third week of life and every 3 week thereafter until NICU discharge	• NC was diagnosed in 20/31 (64%) of infants. • Exposure to furosemide was more common in NC group (65% vs 9%; *p* < 0.001).
Ezzedeen (1988) [33]	cohort	17 premature infants with NC treated with furosemide; 3 premature infants treated with furosemide without NC (control group)	RUS during NICU admission	• No difference in average daily dose or duration of furosemide in NC group compared to control group.
Short (1991) [34]	cohort	79 infants with GA < 32 weeks	Serial RUS	• 21/79 (27%) of infants diagnosed with NC. • No difference in mean total dose of furosemide.
Downing (1991) [35]	cohort	117 infants with BW < 1750 g and BPD treated with furosemide	RUS prior to discharge and in 3–6 month intervals for positive findings of NC/NL	• 20/117 (17%) had evidence of NC/NL on RUS prior to discharge. • Infants maintained on furosemide were more likely to have persistent NC/NL compared to those for whom furosemide was stopped (*p* < 0.001).
Downing (1992) [36]	cohort	27 infants with BW < 1500 g enrolled into 3 groups: 1) not exposed to furosemide (*n* = 7); 2) received furosemide without NC (*n* = 10); and 3) received furosemide with NC (n = 10)	RUS and laboratory testing for glomerular and tubular kidney function	• Infants in group 3 had lower creatinine clearance (reduced glomerular function) and higher tubular dysfunction compared to infants in group 1 and 2.
Stafstrom (1992) [37]	cohort	11 premature infants with post-hemorrhagic hydrocephalus treated with furosemide and acetazolamide	Serial RUS	• 5/11 (45%) infants with evidence of NC. • No correlation between duration of treatment, total dosage of medications, and development of renal calculi.
Pope (1996) [38]	cohort	13 premature infants with NC and exposed to furosemide divided into 2 groups: resolution of NC (*n* = 6) and persistent NC (n = 7).	Serial RUS	• No difference in duration of or cumulative dose of furosemide in infants with resolution of NC compared to those with persistence of NC.
Saarela (1999) [39]	cohort	129 infants with BW < 1500 g	RUS at 2 weeks, 6 weeks, and 3 months of life	• 26/129 (20%) of infants diagnosed with NC. • The mean cumulative doses of furosemide were significantly higher in infants with NC compared to those without NC (19 mg vs 5 mg; p < 0.001).
Schell-Feith (2000) [40]	cohort	215 infants with GA < 32 weeks	RUS at 4 weeks of life and at term	• NC diagnosed in 50/150 (33%) of infants at 4 weeks of life and 83/201 (41%) at term (NS). • At term, furosemide exposure was higher in those with NC (32%) compared to those without NC (18%) (p < 0.001).
Narendra (2001) [41]	cohort	101 infants with GA < 32 weeks or BW < 1500 g	RUS at 1 month of age and at term or NICU discharge	• 16/101 (16%) diagnosed with NC. • The median total dose of furosemide was not significantly different before detection of NC on term RUS and in infants without NC (*p* = 0.75).
Hoppe (2002) [42]	cohort	16 infants with GA < 37 weeks and diagnosed with NC	RUS during NICU admission and every 3–6 months following discharge	• NC persisted in 4/12 (33%) infants who received follow-up. • Infants with resolution of NC received lower dosages of furosemide compared to those with persistent NC (*p* < 0.05).
Hein (2004) [43]	cohort	114 infants with BW < 1500 g divided into 2 groups: 1) NC (*n* = 20); 2) without NC (*n* = 94). 20 infants from control group matched to NC group based on BW and GA.	RUS every 2 weeks during NICU admission	• No difference in duration of furosemide therapy between groups.
Ketkeaw (2004) [44]	cohort	36 infants with GA < 32 weeks and BW < 1250 g	RUS prior to NICU discharge	• 14/36 (39%) were diagnosed with NC. • The mean cumulative dose and mean duration of furosemide was higher in infants with NC compared to those without NC (102 mg vs 32 mg; *p* = 0.001 and 39 vs 7 days; p = 0.001).
Cranefield (2004) [45]	cohort	Cohort of infants enrolled in randomized trial of two regimens of dexamethasone for the prevention of BPD.	RUS on study entry, day of life 28, and at discharge or 36 weeks postmenstrual age	• 15/18 (83%) of infants for whom complete data were available were diagnosed with NC prior to discharge or 36 weeks postmenstrual age. • Furosemide was used infrequently in the trial. 7/8 (88%) of the infants who never received furosemide developed NC.
Gimpel (2010) [46]	cohort	55 infants with GA < 32 weeks and BW < 1500 g	RUS obtained after the first month of life	• 15/55 (27%) of infants were diagnosed with NC. • The strongest independent risk factor for NC was furosemide therapy with cumulative dose > 10 mg/kg (OR 48.1 (95% CI 4.0–585); *p* < 0.01).
Chang (2011) [47]	cohort	102 infants with GA < 34 weeks and BW < 1500 g	RUS at term or prior to NICU discharge	• 6/102 (6%) of infants were diagnosed with NC. • Exposure to furosemide was more common in the NC group compared to the group without NC (33% vs 3%; *p* = 0.027).
Lee (2014) [48]	cohort	52 infants with BW < 1500 g	RUS at 4 and 8 weeks of life	• Exposure to furosemide did not differ significantly between infants with NC and those without NC.
Mohamed (2014) [49]	cohort	97 infants with GA ≤ 34 weeks	RUS at first week of life, at term, and at one year corrected age	• Exposure to furosemide was more common in the NC group compared to the group without NC (50% vs 16%; *p* = 0.003).

Legend: *BW* Birth weight, *GA* Gestational age, *OR* Odds ratio, *CI* Confidence interval, *BPD* Bronchopulmonary dysplasia

A dose-response relationship between furosemide and NC/NL was evaluated in 8 studies [33, 34, 38, 39, 41, 42, 44, 46]. Half of these studies found no association between dose and the development of NC/NL. Two studies found no difference in average daily dose or duration of furosemide between infants with NC and infants without NC [33, 34]. Investigators reported no difference in cumulative dose or duration of treatment between infants with resolution of NC compared to those with persistent findings of NC [38]. An additional study found no difference in median total dose of furosemide before detection of NC and in those infants without NC in another study [41]. In contrast, one study found that infants with resolution of NC received lower daily dosages of furosemide than did those with persistent NC (*p* < 0.05) and another determined that mean cumulative doses of furosemide were significantly higher in infants with NC than in those without NC (18.8 mg vs 5.0 mg; p: 0.001) [39, 42]. A single center study found higher cumulative doses of furosemide in infants with development of NC than in those without NC (mean +/− standard deviation: 102.2 +/− 118.2 mg vs 32.3 +/− 81.1 mg; *p* = 0.001) [44]. Investigators identified exposure to furosemide with cumulative dose > 10 mg/kg was the strongest independent risk factor for NC in a multivariable analysis of premature infants (OR 48.1 (95% CI 4.0–585); *p* < 0.01) [46].

#### Quality assessment

We assessed the quality of each study based on the risk of bias in the seven domains of the ROBINS-I tool for non-randomized studies (Table 6). We determined that one study had a critical risk of bias and resembled a case series in its lack of the use of a control group [30]. The most common reason we considered a study to have a serious risk of bias was the absence of adjustment for severity of illness. Three studies used multivariable analysis to control for the high correlation of cumulative furosemide exposure with birthweight, duration of mechanical ventilation, and severity of BPD, all variables that are independently associated with the development of NC/NL [34, 41, 46]. One study found that furosemide exposure was the strongest independent risk factor for NC, despite controlling for multiple markers of illness severity. However, two studies that considered the temporal relationship between furosemide and NC found no difference in mean dose of furosemide before detection of NC on renal ultrasound in infants who did or did not subsequently develop NC [34, 41]. There was no pattern relating quality to the results of the studies (i.e., both positive and negative studies included a mix of moderate and serious risks of bias).

**Table 6:** Quality assessment of observational studies examining risk of nephrocalcinosis/nephrolithiasis (NC/NL) in premature infants

Study (Year)	Risk of Bias (Low, Moderate, Serious, Critical, No Information)	Comments
Hufnagle (1982) [30]	Critical	Critical risk of bias in confounding domain: no statistical tests performed in the analysis to test association of NC and furosemide. Critical risk of bias in selection of participants into the study: All infants were exposed to furosemide and had NC; lack of control group.
Woolfield (1988) [31]	Serious	Serious risk of bias in confounding domain: no statistical tests performed in the analysis to test association of NC and furosemide.
Jacinto (1988) [32]	Serious	Serious risk of bias in confounding domain: Lower BW and GA associated with outcome (NC), along with exposure to furosemide. Did not control for severity of illness.
Ezzedeen (1988) [33]	Serious	Serious risk of bias in confounding domain: no adjustment for severity of illness; small number of infants in control group.
Short (1991) [34]	Moderate	Multivariate analyses controlling for other risk factors for NC. Dose-response relationship evaluated.
Downing (1991) [35]	Moderate	All infants screened for the outcome had a diagnosis of chronic lung disease; high percentage of follow-up imaging obtained.
Downing (1992) [36]	Moderate	Robust comparators; long-term follow-up.
Stafstrom (1992) [37]	Serious	Serious risk of bias in confounding domain: no statistical tests performed in the analysis to test association of NC and furosemide. No reporting of frequency of NC in infants with post-hemorrhagic hydrocephalus not exposed to furosemide.
Pope (1996) [38]	Moderate	Similar severity of illness in each group; long-term follow up with serial ultrasounds. Dose-response relationship evaluated.
Saarela (1999) [39]	Moderate	Dose-response relationship evaluated.
Schell-Feith (2000) [40]	Moderate	Large sample size. Control group without NC included.
Narendra (2001) [41]	Moderate	Multivariate analyses controlling for other risk factors for NC. Dose-response relationship evaluated.
Hoppe (2002) [42]	Serious	Serious risk of bias in confounding domain: Lack of control group without NC.
Hein (2004) [43]	Moderate	Large sample size with appropriate control groups.
Ketkeaw (2004) [44]	Moderate	Appropriate control group included. Dose-response relationship evaluated.
Cranefield (2004) [45]	Moderate	All infants with comparable severity of illness.
Gimpel (2010) [46]	Moderate	Multivariate analyses controlling for other risk factors for NC. Dose-response relationship evaluated.
Chang (2011) [47]	Serious	Serious risk of bias in confounding domain: no adjustment for severity of illness. Low incidence of NC in sample.
Lee (2014) [48]	Serious	Serious risk of bias in confounding domain: no adjustment for severity of illness.
Mohamed (2014) [49]	Serious	Serious risk of bias in confounding domain: no adjustment for severity of illness.

Legend: *BW* Birth weight, *GA* Gestational age

#### Strength of evidence

We determined that the strength of evidence for the association of NC/NL and furosemide exposure in premature infants is low based on our review of the existing literature. No clinical trials of furosemide have examined the outcome of NC/NL. There is a high risk of bias in the numerous observational studies reviewed as only a few studies accounted for other renal stone-promoting factors, such as concomitant medication use and supplementation of calcium, phosphorus, and vitamin D to reduce the risk of osteopenia of prematurity. The AHRQ classifies evidence as indirect if “it uses intermediate or surrogate outcomes instead of ultimate health outcomes; one body of evidence links the intervention to intermediate outcomes and another body of evidence links the intermediate to most important (health or ultimate) outcomes.” (p. 515) The endpoint of NC/NL is likely a surrogate outcome for chronic kidney disease and cardiovascular disease, which were detected in a minority of infants included in the studies. Although clinical trials are needed to determine the relationship of furosemide and NC/NL, adequate long-term follow-up will also be required to determine any lasting effects of NC/NL on renal and cardiovascular health.

## Discussion

We found no evidence that furosemide exposure increases the risk of SNHL or NC/NL in premature infants. We determined that the strength of evidence for the association of these outcomes with furosemide exposure is low. With the exception of one randomized controlled trial including SNHL as an outcome, all reviewed studies were cohort or case-control studies. These studies’ observational designs left many important potential confounding variables not well-accounted for, such as severity of illness, duration of mechanical ventilation, and concomitant medication exposures. Some of the included cohort studies were of high quality and used multivariable analyses to account for confounding, but randomized controlled trials of furosemide are urgently needed to assess the risks of SNHL and NC/NL in premature infants.

Despite the proposed benefits and biological plausibility of using furosemide to improve respiratory outcomes, the efficacy of furosemide in premature infants has not been established. A Cochrane systematic review examining loop diuretics for preterm infants found no evidence to support an improvement in long-term outcomes, including BPD [50]. However, results from the review indicated that chronic administration of furosemide (i.e., at least 7 days) improves oxygenation and lung compliance in premature infants with established BPD. The review emphasizes the need for randomized clinical trials to assess the effects of furosemide administration on morbidity and mortality.

Notwithstanding the absence of data supporting the efficacy of furosemide, the medication is commonly used by clinicians in the neonatal intensive care unit (NICU) with the intention to improve oxygenation and wean respiratory support in premature infants. A cohort study from more than 300 NICUs in the U.S. found that furosemide was the fifth most common drug used between the years 2005 to 2010 in premature infants with birth weights < 1000 g, with approximately 50% of these infants exposed to at least one dose of furosemide during their initial hospitalization [51]. Although other diuretic medications are used in the NICU for similar indications, furosemide is by far the most commonly used diuretic in the NICU, accounting for 93% of diuretic use [52]. Furosemide is also variably used in the NICU setting. A cohort study of infants with birth weights < 1500 g from more than 200 U.S. NICUs over a 15 year period found considerable variability in the percentage of infants exposed to at least one dose of furosemide at each particular site, with a median exposure by site of 33% and a range of 0 to 75%. The observations from this study emphasize the lack of a universally accepted standard governing when to expose an infant in the NICU to furosemide and indicate that at some centers, no exposure to furosemide is an option [52].

Additional potential adverse outcomes of furosemide use in premature infants include metabolic bone disease (osteopenia) of prematurity and electrolyte abnormalities related to urinary loss of sodium, chloride, and calcium [53–55]. As with other adverse outcomes, the etiology of metabolic bone disease in premature infants likely has many causes, and infants with severe illness often have multiple risk factors such as insufficient phosphorus intake, vitamin D deficiency, prolonged immobilization, mechanical ventilation, and exposure to steroids and antibiotics [56–58]. However, there is a lack of consensus on the definition of metabolic bone disease of prematurity, with some definitions relying on serum mineral levels and others based on radiographical findings [59]. As a result, this review did not focus on metabolic bone disease of prematurity as an outcome of interest in premature infants exposed to furosemide.

Existing safety data on the use of furosemide in adults and children cannot be extrapolated to premature infants due to higher extracellular fluid volume per unit body weight, immature hepatic and renal function, and a more permeable blood-brain barrier in premature infants, variables which can alter the rates of drug absorption, distribution, metabolism, and elimination [60]. Four studies of furosemide pharmacokinetics in premature infants demonstrate significant variability in the volume of distribution, clearance, and half-life of the drug based on gestational age at birth, birth weight, and postnatal age [61–64]. The limited available data on pharmacokinetics and safety have led to a growing recognition of the need for clinical trials in premature infants to determine the safety and efficacy of therapeutic agents, such as furosemide [65, 66].

A phase II clinical trial by the Pediatric Trials Network is underway to better understand the safety of furosemide in premature infants at risk of BPD [67]. This trial will enroll premature infants born < 29 weeks’ gestation and receiving positive airway pressure or mechanical ventilation on 7–28 days postnatal age. The study design includes a dose escalation schedule with three cohorts randomized to placebo or furosemide. Primary outcomes will include safety information on SNHL (based on BAER prior to discharge) and NC/NL determined by serial renal ultrasounds. Secondary outcomes will assess effectiveness of early furosemide in the prevention of BPD or death. The dose escalation design of the study will allow for evaluation of a dose-response relationship of furosemide and the outcomes of SNHL and NC.

Premature infants, particularly those born small for gestational age, are at an increased risk for cardiovascular and renal disease in later life, likely due to a combination of genetic and environmental factors [68]. It is important to understand whether furosemide exposure, with or without evidence of NC/NL, contributes to the development of chronic kidney disease in these infants. Future studies investigating the safety of furosemide in premature infants will need to consider long-term surveillance of renal disease, particularly in those who develop NC/NL. The study design of trials investigating furosemide in the neonatal population should incorporate serial renal sonography, blood pressure measurements, urinalysis for proteinuria, and serum creatinine, where appropriate, to determine renal function over time.

This systematic review was limited by the inclusion of only one randomized controlled trial. The remaining studies were cohort and case-control studies, which by the nature of their study design are unable to demonstrate a causal role. While many randomized controlled trials of furosemide in premature infants have been performed, very few included data on safety outcomes, which were the subject of this review. In addition, the search strategy was limited to studies published in English may not have included all available studies; for instance, we found that some studies included from our review of reference lists used variable spellings of “furosemide.” The field of neonatology has changed dramatically over the past 20 years with the introduction of antenatal steroids, surfactant replacement therapy, and non-invasive ventilation strategies. These practices have resulted in significantly improved survival and reduced morbidities in premature infants [69, 70]. Therefore, results from studies included in this review which were performed decades ago may not be applicable to infants born in the current era. Finally, we were unable to perform a meta-analysis of the included observational studies due to a lack of uniformity in outcome definitions, population characteristics, and length of follow-up.

## Conclusions

This systematic review of the safety of furosemide in premature infants focuses on the outcomes of SNHL and NC/NL. Irrespective of the efficacy of furosemide in premature infants, the existing literature on safety outcomes depends almost entirely on observational data. Furthermore, few studies used analytic approaches to control for confounding causes of additional risk of SNHL and NC/NL on premature infants exposed to furosemide. The strength of evidence for the association of SNHL and NC/NL and furosemide is therefore determined to be low. Further randomized controlled trials with robust safety measures, such as the ongoing PTN trial, are urgently needed to assess the safety of furosemide in premature infants.

## Additional file

Additional file 1: Data Abstraction Form. (DOCX 14 kb)

## Abbreviations

ABR: Auditory brain stem response
AHRQ: Agency for Healthcare Research and Quality
BAER: Brainstem auditory evoked response
BPD: Bronchopulmonary dysplasia
FDA: Food and Drug Administration
NC: Nephrocalcinosis
NICU: Neonatal intensive care unit
NL: Nephrolithiasis
OAE: Otoacoustic emission test
PTN: Pediatric Trials Network
SNHL: Sensorineural hearing loss
