# Correction to: Association between furosemide in premature infants and sensorineural hearing loss and nephrocalcinosis: a systematic review

**DOI:** 10.1186/s40748-019-0108-6

**Published:** 2019-07-31

**Authors:** Wesley Jackson, Genevieve Taylor, David Selewski, P. Brian Smith, Sue Tolleson-Rinehart, Matthew M. Laughon

**Affiliations:** 1 Division of Pediatrics, University of North Carolina at Chapel Hill, UNC Hospitals 101 Manning Dr. 4th Floor, Chapel Hill, NC CB 7596 USA; 2 Division of Nephrology, Department of Pediatrics and Communicable Diseases, C.S. Mott Children’s Hospital, University of Michigan, Ann Arbor, MI USA; 3 Duke Department of Pediatrics, Duke University Medical Center, Durham, NC USA; 4 Gillings School of Global Public Health, University of North Carolina at Chapel Hill, Chapel Hill, USA

**Correction to: Matern Health Neonatol Perinatol (2018) 4:23**

**https://doi.org/10.1186/s40748-018-0092-2**

Following publication of the original article [1], the authors notified us that they would like to acknowledge Anjali Gupta for her contribution to the background for this systematic review.
