# Supplementary material for: Association between furosemide in premature infants and sensorineural hearing loss and nephrocalcinosis: a systematic review
Source: Matern Health Neonatol Perinatol. 2018 Nov 19;4:23. doi: 10.1186/s40748-018-0092-2 (PMC6240934; doi:10.1186/s40748-018-0092-2)
Supplement: Supplementary file 1 — Data Abstraction Form. (DOCX 14 kb) [file 40748_2018_92_MOESM1_ESM.docx]

**Additional file 1**

**Data Abstraction Form**

Author (Year):

Reference:

Eligibility

1. Was the study originally published in English? Y/N/Unclear
2. Did the study use human participants? Y/N/Unclear
3. Does the study include premature infants (< 37 weeks GA)? Y/N/Unclear
4. Were participants exposed to at least one dose of furosemide (enteral or intravenous)?

Y/N/Unclear

1. Does the study examine the risk of hearing loss or nephrocalcinosis/nephrolithiasis in premature infants exposed to at least one dose of furosemide?

Y/N/Unclear

Study Characteristics (if eligible)

1. Study Design:
2. Sample size:
3. Gestational age and/or birth weight criteria:
4. Control group (if applicable):
5. Outcome(s) examined:
6. Duration of follow-up:
7. Summary of results:
